# Optimising care and follow-up of adults with achondroplasia

**DOI:** 10.1186/s13023-022-02479-3

**Published:** 2022-08-20

**Authors:** Svein Fredwall, Yana Allum, Moeenaldeen AlSayed, Inês Alves, Tawfeg Ben-Omran, Silvio Boero, Valerie Cormier-Daire, Encarna Guillen-Navarro, Melita Irving, Christian Lampe, Mohamad Maghnie, Klaus Mohnike, Geert Mortier, Sérgio B. Sousa, Michael Wright

**Affiliations:** 1 TRS National Resource Centre for Rare Disorders, Sunnaas Rehabilitation Hospital, Nesodden, Norway; 2 Independent, Perth, WA Australia; 3 Department of Medical Genetics, King Faisal Specialist Hospital and Research Center, Riyadh, Kingdom of Saudi Arabia; 4 Faculty of Medicine, Alfaisal University, Riyadh, Kingdom of Saudi Arabia; 5 ANDO Portugal, Evora, Portugal; 6 Division of Genetics and Genomic Medicine, Sidra Medicine and Hamad Medical Corporation, Doha, Qatar; 7 Pediatric Orthopaedic and Traumatology Unit, Istituto Giannina Gaslini, Genoa, Italy; 8 Centre of Reference for Constitutional Bone Diseases (MOC), Department of Clinical Genetics, Paris Centre University, INSERM UMR 1163, Imagine Institute, Necker-Enfants Malades Hospital, Paris, France; 9 Medical Genetics Section, Department of Pediatrics, Virgen de La Arrixaca University Hospital, IMIB-Arrixaca, University of Murcia-UMU, Murcia, Spain; 10 CIBERER-ISCIII, Madrid, Spain; 11 Department of Clinical Genetics, Guy’s and St Thomas’ NHS Foundation Trust, London, UK; 12 Clinic of Neuropediatrics, Epileptology and Social Pediatrics, University Hospital Giessen and Marburg, Giessen, Germany; 13 Department of Pediatrics, IRCCS Istituto Giannina Gaslini, 16147 Genoa, Italy; 14 Department of Neuroscience, Rehabilitation, Ophthalmology, Genetics, Maternal and Child Health, University of Genova, 16147 Genoa, Italy; 15 Central German Competence Network for Rare Diseases (ZSE), Universitätskinderklinik, Otto-Von-Guericke Universität, Magdeburg, Germany; 16 KU Leuven, Department of Human Genetics and Centre of Human Genetics, UZ Leuven, Leuven, Belgium; 17 Medical Genetics Unit, Hospital Pediátrico, Centro Hospitalar E Universitário de Coimbra, Coimbra, Portugal; 18 Faculty of Medicine, University Clinic of Genetics, Universidade de Coimbra, Coimbra, Portugal; 19 Newcastle Hospitals, Newcastle, UK

**Keywords:** Achondroplasia, European Achondroplasia Forum, Adult, Transition, Guiding principles, Management, Recommendations

## Abstract

**Background:**

Achondroplasia is a genetic condition that can cause complications across the lifespan. While complications in childhood are well documented, the natural history of achondroplasia in adults has, until recently, been relatively lacking, and little is known about the care they receive or how they access it. The European Achondroplasia Forum undertook two exploratory surveys, one for healthcare professionals (HCPs) and one for patient advocacy group (PAG) representatives, to gain an understanding of current practices of the transition process of individuals with achondroplasia from paediatric to adult services and how adults perceive their care.

**Results:**

Most HCP respondents followed up more children than adults, and 8/15 responded that individuals did not transition to an adult multidisciplinary team (MDT) after paediatric care. Of 10 PAG respondents, none considered the experience of transition to adult services as good or very good and 50% considered it to be poor or very poor. A total of 64% (7/11) described the coordination of transition to adult services as “Not satisfactory” or “Poor”. HCPs and PAG representatives largely agreed on the core specialists involved in adult care (orthopaedic surgeons, physiotherapists, rehabilitation specialists, rheumatologists, clinical geneticists). However, there was a discrepancy in the understanding of healthcare needs outside of this, with PAG representatives selecting neurosurgeons and genetic counsellors, while HCPs selected pulmonologists and obstetricians/gynaecologists. There was agreement between HCP and PAG respondents on the key barriers to effective care of adults with achondroplasia, with lack of an adult MDT, lack of interest from individuals in accessing care, and less experience in adult than paediatric MDTs ranking highly.

**Conclusions:**

This study indicates that the care and follow up of adults with achondroplasia is challenging. Individuals are often lost to, or decline, follow up as they leave paediatric care, and it is largely unknown how, where, and why adults with achondroplasia access care later in life. Lifelong, multidisciplinary specialist care led by an identified physician should be accessible to all individuals with achondroplasia. It is important to ensure barriers to optimal care are addressed to enable access to appropriate care for all individuals with achondroplasia.

**Supplementary Information:**

The online version contains supplementary material available at 10.1186/s13023-022-02479-3.

## Background

Achondroplasia is an autosomal dominant genetic condition that can cause complications across the lifespan, thereby requiring lifelong management [1–5]. It is caused by a recurrent pathogenic variant in the fibroblast growth factor receptor 3 (*FGFR3*) gene [6, 7]. Many complications such as foramen magnum stenosis, upper airway obstruction, and thoracolumbar kyphosis occur in infancy and early childhood, and these are well documented [3–5]. In comparison, the natural history in adults with achondroplasia has, until recently, been relatively lacking [8–12]. An increasing number of studies have emerged in recent years describing potential medical complications and impact on psychosocial health in adults. Among the most severe medical complications in adulthood are symptomatic spinal stenosis, obstructive sleep apnoea, hearing loss, hypertension, and obesity [13–19]. Reduced physical functioning, impaired ability to perform activities of daily living, reduced work participation, chronic pain, depression, anxiety, and low self-esteem are other challenges commonly reported in the adult achondroplasia population [14, 20–23]. The prevalence of complications in adulthood and the subsequent impact on physical functioning and daily life are significant [14]. With the medical complications of achondroplasia extending into adulthood, and differing to those seen in childhood, management throughout the life course is warranted.

Two sets of recommendations were recently published on the management of individuals with achondroplasia; the first by our group, the European Achondroplasia Forum (EAF) [1], and the second, a consensus statement from an international group of 55 healthcare professionals managing achondroplasia and patient representatives [2]. Both guidelines advocate for the management of achondroplasia throughout the life course to address the varied and multifaceted complications experienced by individuals with this condition [1, 2]. The EAF Guiding Principles for Management state that “*achondroplasia is a lifelong condition requiring lifelong management by an experienced MDT, led by physicians/clinicians experienced in achondroplasia management*” [1], while Savarirayan et al. state that *“… it is critical to adopt a multidisciplinary and pro-active approach to the clinical and psychosocial care of individuals with ACH throughout their life*”. [2]

Most specialists in achondroplasia are based in paediatrics, and in many cases multidisciplinary team (MDT) management is in place in the paediatric setting [4, 24]. However, as with many chronic conditions [25], the period of transition in achondroplasia management from paediatric to adult care is not well established and individuals are lost to follow up during this period. This loss to follow up reduces the understanding of achondroplasia natural history in adults and leads to a paucity of specialists with experience and understanding of the care needed by adults [12]. Little is known about who manages the care of adults with achondroplasia and how that care is structured. The needs and expectations of adults with achondroplasia regarding healthcare provision after childhood is also largely unknown.

The EAF (European Achondroplasia Forum) is an independent network of specialists in achondroplasia management, representative of the clinical community, with the aim of improving overall care for individuals with achondroplasia through collaboration and cross-country sharing of best practices. The Steering Committee includes representation from across Europe and the Middle East and is comprised of a general practitioner specialised in the care of adults, clinical geneticists, paediatric endocrinologists, orthopaedic surgeons, and a neuropaediatrican. The EAF holds open workshops, which are advertised through the contacts of the Steering Committee, the EAF website (www.achondroplasiaforum.com) and social media. The EAF seeks expert input, including from patient advocacy groups, representative of large groups of individuals with achondroplasia, to ensure all opinions are included in their discussions.

With international consensus on the management of achondroplasia [2], the EAF sought to establish current practice in relation to the largely unknown areas of transition from paediatric to adult care, and the management of achondroplasia in adults. The aims of the study were to gain an understanding of current practices in specialist centres in Europe, identify the gaps in our knowledge, and the key barriers to effective transition of care and care of adults with achondroplasia, as well as establishing areas for future research.

## Methods

Two exploratory surveys were developed to gain a greater understanding of the experiences of specialist centres and individuals with achondroplasia of the transition from paediatric to adult services and the care of adults with achondroplasia. One survey was designed to gain the perspective of healthcare professionals (HCPs) (Additional file 1), and the other the perspective of patient advocacy group (PAG) representatives (Additional file 2). The exploratory surveys were developed by a member of the EAF Steering Committee (SF) and a PAG representative (IA); both are co-authors of this paper. Detailed definitions of wording, such as “Satisfactory” or “Poor” were not provided in the survey, so a level of personal interpretation was permitted. The exploratory surveys were designed to capture a snapshot of current practices in responding centres, and the lived experience of individuals with achondroplasia presented by a PAG representative; as such, the surveys were not validated. The HCP survey was distributed via email to the EAF Steering Committee, and the PAG survey was distributed via email to a list of contacts from Patient Advocacy Groups across Europe, provided by the PAG representative who supported the development of the survey (IA). All recipients were asked to circulate the survey to their colleagues and personal networks. We cannot therefore quantify how many people received the survey. Not all those who received the survey completed it.

The results of the exploratory surveys were collated and presented at an online open workshop to a group of PAG representatives and HCPs experienced in the management of achondroplasia. The workshop was attended by the EAF Steering Committee and invitations were extended to their colleagues and personal networks, as well as to all the PAG representatives who were sent the exploratory survey. The participants in the session discussed the results of the surveys, assessed barriers and proposed strategies to improve the transition process from paediatric to adult care, and the care of adults with achondroplasia. A total of 28 attendees from 14 countries participated. Attendees included clinical geneticists, paediatric endocrinologists, an orthopaedic surgeon, a neuropaediatrician, primary care physicians, patient advocates, an orthodontist, and a rheumatologist. There was representation from Australia, Belgium, France, Germany, Hong Kong, Italy, Saudi Arabia, Malaysia, Norway, Portugal, Qatar, Spain, Sri Lanka, and the United Kingdom. The session was also attended by HCP experts in the management of another chronic condition, who presented their experience of transition from paediatric to adult care in haemophilia.

## Results

### Healthcare professionals

**Fig. 1:**
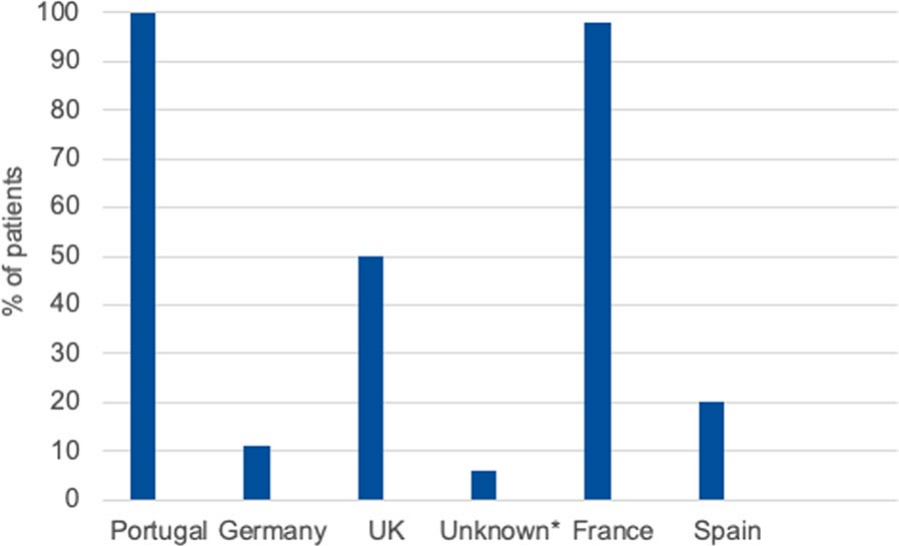
Patients for whom a structured transition process is followed. *Respondent did not specify their country

Data was collected from 16 respondents from 10 countries (France, Germany, Italy, Belgium, Qatar, Kingdom of Saudi Arabia, Norway, Portugal, Spain, and the United Kingdom). The majority of respondents were medical or clinical geneticists (11/16), all of whom were working in a university/teaching hospital, specialist achondroplasia centre or academic institution. There were two paediatric endocrinologists, also based in academic institutions, a general practitioner based at a Resource Centre for Rare Disorders and a paediatric orthopaedic surgeon based at a paediatric hospital. Respondents managed a greater number of paediatric than adult patients, except for one who had more adults in their care. Six respondents have structured transition from paediatric to adult achondroplasia services at their centre (Table 1). However, this is generally not followed for all patients (Fig. 1), and the process of transition varies widely (Table 2).

**Table 1:** HCP perspectives of the structure and management of achondroplasia care in adulthood

Question	No. of responses*
Is there a structured transition process from paediatric to adult services?	
Yes	6
No	9
Do patients leaving/attending your centre transition to an MDT for management of achondroplasia in adulthood?	
Yes	6
No	8
I don’t know	1
Is there a lead clinician in the management of adults with achondroplasia?	
Yes	11
No	3
I don’t know	1
If yes, who is the lead clinician?	
Endocrinologist	4
Rheumatologist	3
Clinical geneticist	4
Orthopaedic surgeon	0
Genetic counsellor	0
Primary care physician	1

*Not all respondents answered all questions

**Table 2:** Transition processes

UK	“There is no structured transition process”
France	“At 16–17 years, there is a clinic with the adult rheumatologist in our paediatric centre. At 18 years there is a multidisciplinary clinic in the adult hospital with the paediatric team (geneticist and orthopaedist) and the adult team (rheumatologist and orthopaedic surgeon)”
Norway	“[there is] no transition as we [The National Resource Centre] offer lifelong care”
Spain	“The core group in the skeletal dysplasia clinic (Medical Genetics, RHB and traumatologist) follow paediatric and adults”

Please note, these are the opinions of individual survey respondents, and may not reflect practices across their country

**Fig. 2:**
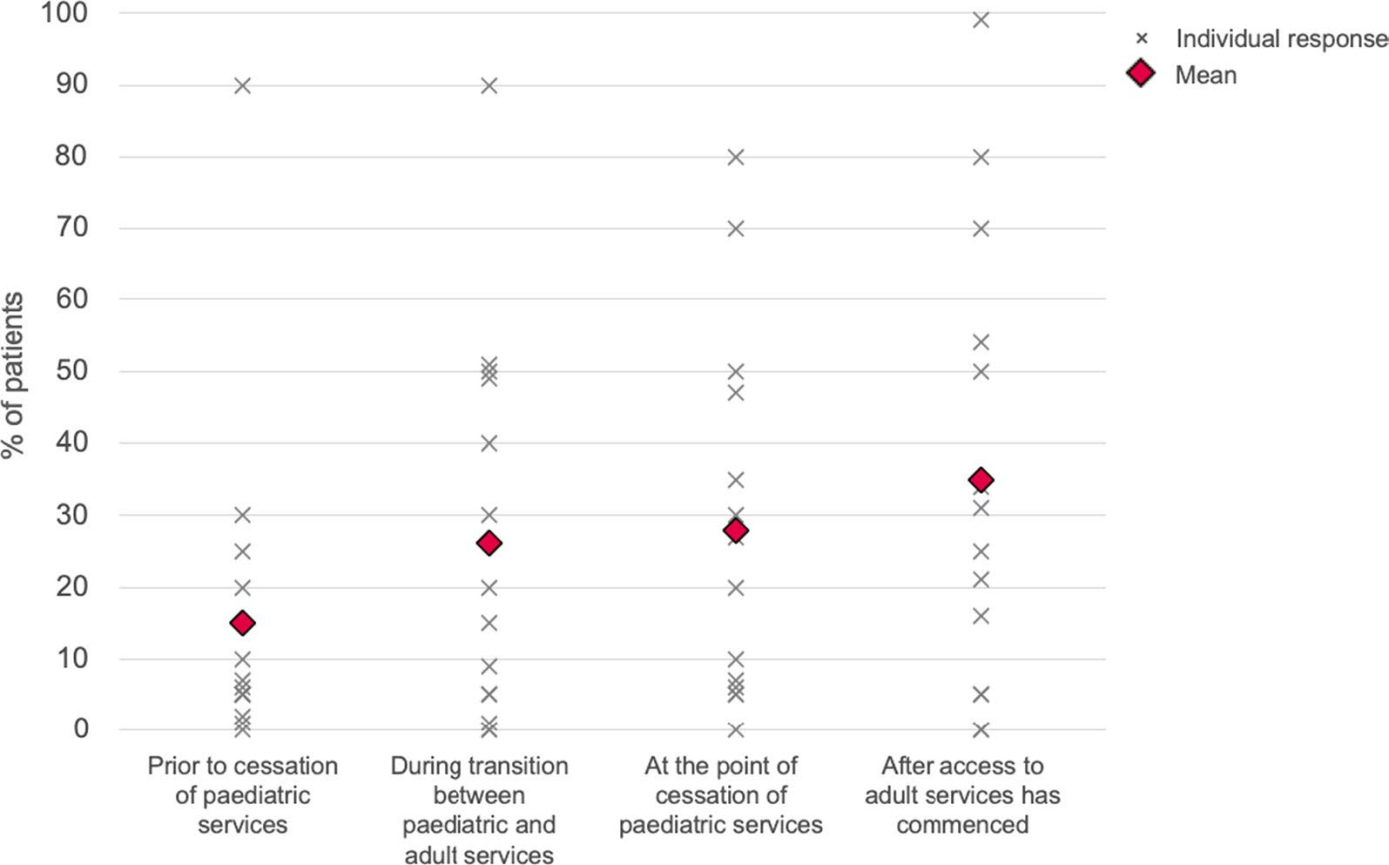
Proportion of patients lost to follow-up, by stage. Figures provided by respondents are estimates

Eight of 15 respondents answered that individuals with achondroplasia do not transition to an adult MDT, and one did not know whether they did or not (Table 1). Our results also showed that individuals are increasingly lost to, or decline follow up after leaving paediatric care (Fig. 2).

**Fig. 3:**
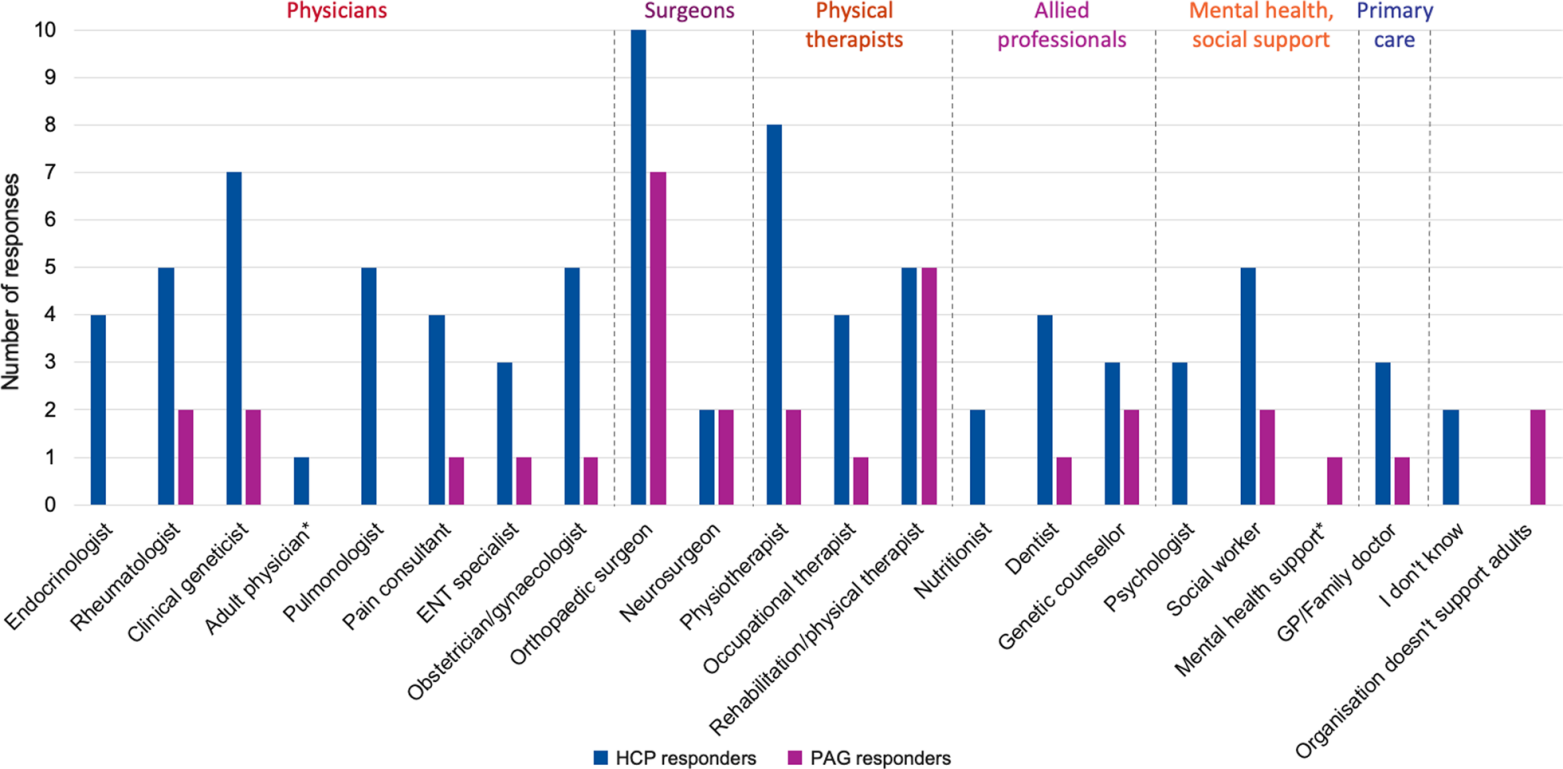
Specialties that are part of an adult MDT managing achondroplasia. *Adult physician added as ‘other’ in HCP survey by one respondent; Mental health support added as ‘other’ in PAG survey by one respondent. ENT, Ear, nose and throat. Two PAG respondents stated that their group did not support adults

There was a wide variety of specialties identified as managing care in adults with achondroplasia, including physicians, surgeons, physical therapists, allied professionals, mental health support and primary care (Fig. 3). Most respondents (11/16) were able to identify a lead physician (Table 1).

**Fig. 4:**
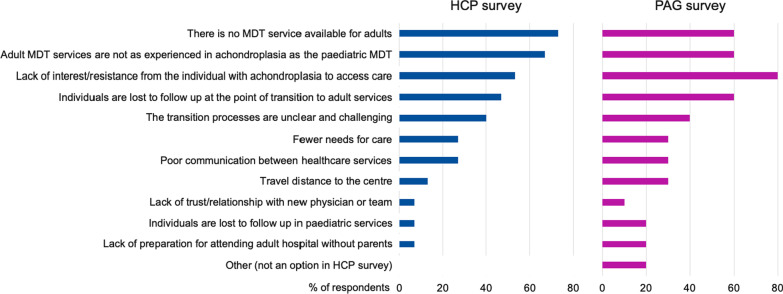
Key barriers to effective management in adulthood and transition to adult care

When assessing the main barriers to effective care for adults with achondroplasia, the most frequent responses were “There is no MDT service available for adults”, “Adult MDT services are not as experienced in achondroplasia management as the paediatric MDT” and “Lack of interest/resistance from the individual with achondroplasia to access care” (Table 3, Fig. 4).

**Table 3:** Key barriers to effective transition from paediatric to adult services and management in adulthood

Question n, %	HCP (n = 15*)	PAG (n = 10*)
There is no MDT service available for adults	11 (73)	6 (60)
Adult MDT services are not as experienced in achondroplasia management as the paediatric MDT	10 (67)	6 (60)
Lack of interest/resistance from the individual with achondroplasia to access care	8 (53)	8 (80)
Individuals are lost to follow up at the point of transition to adult services	7 (47)	6 (60)
The transition processes are unclear and challenging	6 (40)	4 (40)
Fewer needs for care	4 (27)	3 (30)
Poor communication between healthcare services	4 (27)	3 (30)
Travel distance to the centre	2 (13)	3 (30)
Lack of trust/relationship with new physician or team	1 (7)	1 (10)
Individuals are lost to follow up in paediatric services	1 (7)	2 (20)
Lack of preparation for attending adult hospital without parents	1 (7)	2 (20)
Poor communication between the individual with achondroplasia (or family) and the MDT	0	0
Other		2 (20)

*Respondents could select more than one answer

### Patient advocacy group representatives

There were 19 respondents to the PAG survey, from 11 countries (Czech Republic, France, Germany, Ireland, Italy, Norway, Portugal, Russia, Serbia, Spain, and the United Kingdom). All responders reported that there was at least one centre of excellence/referral centre for achondroplasia in their country (Table 4). However, three did not know how many centres of excellence there were. A majority (14/16) responded that a paediatric MDT exists in some/all centres of excellence, and this figure dropped (8/12) for adult MDTs (Table 4).

**Table 4:** PAG perspectives of the structure and management of achondroplasia care

Question	No. of responses*
How many centres of excellence/referral centres for skeletal dysplasia/rare bone conditions (including achondroplasia) are there in your country?	
0	0
1	2
2	5
3	1
≥ 4	7
I don’t know	3
Are paediatric multidisciplinary teams for achondroplasia available in centres of excellence/referral centres?	
Yes, in all	9
Yes, in some	6
No	0
I don’t know	1
Are adult multidisciplinary teams for achondroplasia available in centres of excellence/referral centres?	
Yes, in all	2
Yes, in some	6
No	4
I don’t know	3
Do healthcare teams or processes to facilitate the transition of children with achondroplasia from paediatric to adult services exist in your country?	
Yes, in all	0
Yes, but only in centres of excellence	9
Not that I know of	6

*Not all respondents answered all questions

**Fig. 5:**
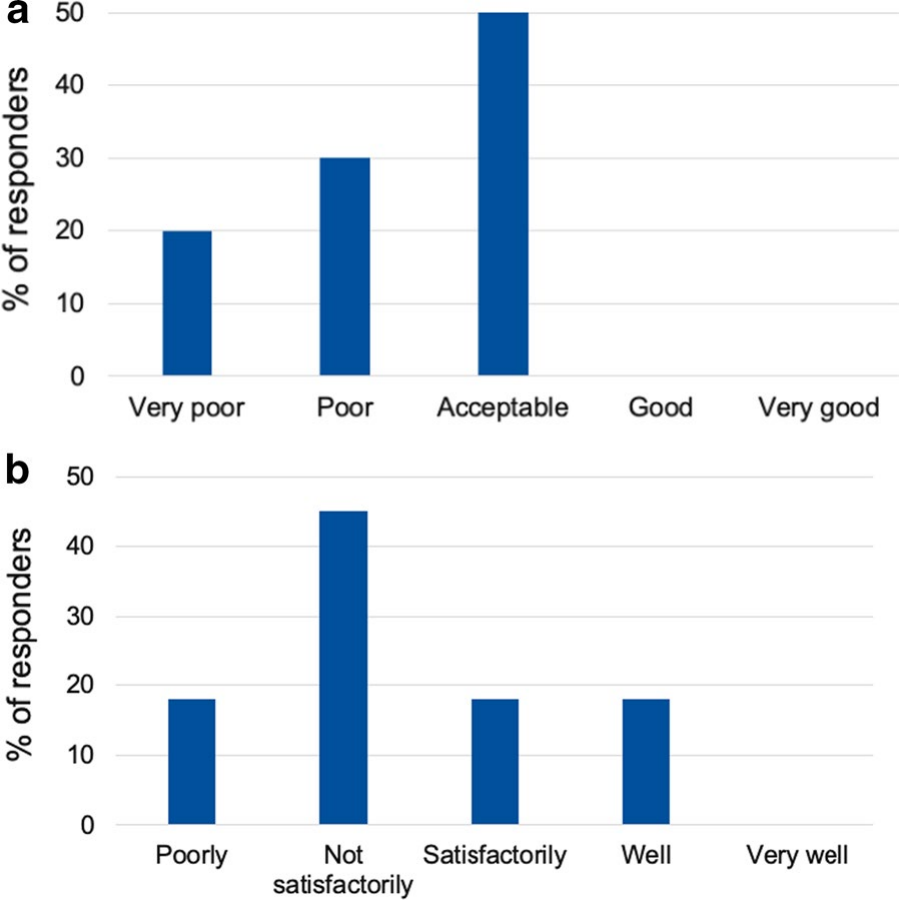
Patient opinion on the process of transition from paediatric to adult services. **a** Overall experience. **b** How well coordinated the process is by healthcare professionals

Results obtained indicate that processes to facilitate the transition from paediatric to adult care exist, but only in centres of excellence (Table 4). Six respondents did not know if there were any transition processes to adult services in their country. Of 10 respondents, none considered the experience of transition to adult services to be good or very good and 50% considered it to be poor or very poor (Fig. 5). A total of 64% (7/11) described the coordination of transition to adult services as “Not satisfactory” or “Poor” (Fig. 5).

The patient representatives identified a wide variety of specialties involved in managing care of adults with achondroplasia (Fig. 3). The main barriers to effective transition of care and follow up of adults identified by the patient representatives were “Lack of interest/resistance from individuals with achondroplasia to access care”, “There is no MDT service available for adults”, “Adult MDT services are not as experienced in achondroplasia management as the paediatric MDT” and “Individuals are lost to follow up at the point of transition to adult services” (Table 3, Fig. 4).

## Discussion

To be able to improve care for adults with achondroplasia, it is important to understand current practices and care provision from the perspective of both the healthcare provider and the recipients of care. Increasing understanding of current practices will enable consideration of how progress can be made towards improved and needs-centred care for adults with achondroplasia. We undertook this study to improve our understanding of current practices in light of recent recommendations advocating for lifelong care by an MDT [1, 2]. This study demonstrates that coordinated MDT care for adults with achondroplasia is not common practice in most countries that responded to the survey. The transition from paediatric to adult care is generally not well coordinated, and many patients are lost to follow up. Main barriers to effective care reported by the HCPs and PAG representatives included reduced availability of MDT services for adults with achondroplasia, adult care is generally not well coordinated, adult MDT service is not as experienced in achondroplasia management as the paediatric MDT, and lack of interest/resistance from the individual with achondroplasia to access care.

The EAF Guiding Principles of Management and an International Consensus Statement both advocate for lifelong MDT management [1, 2], however the results of our survey indicate that current practice for the care of adults with achondroplasia is suboptimal in many countries. Among the centres who responded, coordinated MDT care for adults with achondroplasia does not always exist in practice. It was also observed that there was reduced availability of MDT services for achondroplasia as individuals transitioned from the paediatric to the adult setting, and adult care was generally not well coordinated. Our results indicate that, to the knowledge of the respondents, in some countries in Europe individuals are increasingly lost to follow up after paediatric care or following the transition to adult services. The process of transition is inadequate, with 50% of PAG responders describing their experience as poor or very poor, and none describing the experience as good. There is little way of knowing where and how individuals access care later in life.

The period of transition to adult care occurs at a time of great change in a young person’s life, when their concerns and priorities are changing [25, 26]. There is evidence to suggest that health behaviours established in adolescence persist into adulthood [27], so this is an optimal time to engage young people in their own care. The PAG representatives identified disinterest in clinical care as a barrier for adolescents and young people, in addition to the possibility that they are likely to be fairly healthy at the time in their lives when transition to adult care takes place. They acknowledged that young people may also feel fatigued by the extent of care they have received as children and feelings of embarrassment may begin to emerge in discussing their condition. As the roles of the parents and young people evolve, with individuals wanting increasing independence over managing their care [25], young adults may wish to take ownership of their condition without their parents attending appointments [25, 26].

A key barrier to effective transition and care of adults with achondroplasia identified by both the PAG representatives and the HCPs was a resistance to access care as an adult. Healthcare utilisation can be affected by many factors including availability, proximity, timeliness and convenience, whether patients can receive the care they need, and whether there are providers to meet those needs [28]. The HCP and PAG representatives identified that the lack of availability of coordinated care, as well as the unsatisfactory transition to adult services, lack of understanding of the natural progression of achondroplasia and lack of clarity on where the experts in achondroplasia are could all be contributing factors to the resistance of adults to access care. The level of expertise at the point of access to care is also a concern [25], with one PAG representative commenting that “If we don’t go to someone with experience in skeletal dysplasia, most of the appointment is spent explaining skeletal dysplasia—rather than the health issue being experienced.” Being listened to and understood is key to young people [26]. With limited access to specialists in the management of achondroplasia during the transition period and into adulthood, individuals may feel they are not transitioning to a valuable service.

The PAG respondents and HCPs at the EAF open session outlined several key areas of importance in the care of adults with achondroplasia. These were: the ability to access care independently (e.g., without parental assistance), increased health literacy to understand their own condition and complications that may arise through the lifespan, HCP education, clear identification of specialists with experience in the follow up and care of adults with achondroplasia, and clearly identified referral pathways to access specialists when needed. Self-healthcare management was presented as a real-life example by PAG representatives. This involves an individual being responsible for their own care and holding their own records, accessing healthcare services when needed and taking their own records to appointments. Some of the PAG representatives attending the EAF open session already hold their own records but stressed the importance of good referral pathways for this to be effective.

There were some differences in the perception of healthcare provision between the HCP and PAG respondents. The HCPs and PAG representatives largely agreed on the most frequently accessed specialists in adulthood, with orthopaedic surgeons, physiotherapists, rehabilitation specialists, rheumatologists, clinical geneticists, and social workers ranking highly in both surveys. The HCPs also ranked pulmonologists and obstetricians/gynaecologists highly, whereas the PAG representatives prioritised neurosurgeons and genetic counsellors, indicating a discrepancy in the understanding of care needed outside of the core specialties.

Given the wide variety of specialities involved in the care of adults with achondroplasia, a lead physician experienced in achondroplasia is needed to coordinate care [1, 2]. The lead physician and where they are based (e.g., primary care, hospital, centre of excellence) will vary by country and healthcare system. While an adult MDT may not be based in a single clinic, appropriate management of all the issues associated with achondroplasia throughout the life stages is required for optimal outcomes [2]. There must be sufficient expertise in the core MDT, with access to further specialists when required. As per the recommendations [1, 2], those managing achondroplasia must be experienced in the condition; a family doctor or general practitioner is unlikely to have sufficient knowledge to access the full range of services required by an individual with achondroplasia. In some systems, specialist nurses could be a pivotal contact between the individual and the MDT.

As the participating centres are Centres of Excellence or are led by specialists in the management of achondroplasia, it can be assumed that these centres would provide optimal transition processes and management of achondroplasia in adults. However, the results of our survey indicate that, despite this and despite the recent guidelines, current practice for the care of adults with achondroplasia in the responding centres is suboptimal. It can therefore be assumed that care of young people and adults with achondroplasia outside of these centres is also lacking.

### Limitations

There are several limitations to our study. The HCP survey was circulated to members of the EAF Steering Committee and their colleagues, introducing inclusion bias as most respondents were specialists in achondroplasia management and are based in university/teaching hospitals. Even so, where positive bias would have been expected, the results, even in specialist referral centres, show there is frequently a lack of structured care for adults with achondroplasia. All but one of the HCP respondents managed a greater number of children than adults, although most had some adult patients at their centre. The PAG survey was circulated to more than 20 PAG organisations in Europe supporting individuals with achondroplasia and their families, and while the respondents are representative of different patient communities, it is likely that some individual views were expressed. In addition, not all respondents answered all questions fully. Anecdotally, advocacy groups serve a minority of individuals, and many groups mostly support families with young children as these are often more interested in updated information and communication. Adults with achondroplasia are not involved in advocacy organisations as often, possibly because they have not been in contact with a PAG as children, and/or may not perceive a need for support. As such, a comprehensive picture of the needs of the broad patient community and individual experiences of adults were not fully gathered. Based on this work, conclusions that apply to the whole achondroplasia community in Europe cannot be drawn. However, with some well-known European achondroplasia centres taking part, and some of the most vocal and active patient groups from European countries represented, this was an important initial step to begin increasing our understanding of the wider picture for young people transitioning from paediatric to adult services, and for adults with achondroplasia.

## Conclusions

International recommendations advocate for management of achondroplasia by a MDT through the life stages [1, 2]. However, our results demonstrate that many individuals with achondroplasia do not have access to the care that recent best practice guidelines recommend. The period of transition is an area of care that is particularly lacking, and many patients are lost to follow up. Reduced availability of MDT services for adults with achondroplasia, adult MDT service not being as experienced in achondroplasia management as the paediatric MDT, and lack of interest/resistance from the individual with achondroplasia to access care, were key barriers identified to the effective transition and management of achondroplasia in adults.

This is a complex area, and this study has demonstrated that as a community we are a long way from a standardised approach. Further work is needed to fully understand the patient journey through life with achondroplasia. Greater patient and HCP education and improved communication is required, and the collection of natural history data is essential.

It is important to understand current practices and care provision from the perspective of both the healthcare provider and the recipients of care. It is also crucial to ensure that barriers to optimal care are identified and addressed. As our understanding of this little-known area of achondroplasia care grows, and as the care of adults with achondroplasia is increasingly on the agenda, progress can be made towards improved and needs-centred care for adults with achondroplasia.

## Supplementary Information

**Additional file 1.** Managing Achondroplasia into Adulthood – Healthcare Professionals Survey.

**Additional file 2.** Managing Achondroplasia into Adulthood – Patient Survey.

## Data Availability

Not applicable.

## Abbreviations

EAF: European Achondroplasia Forum
HCP: Healthcare professionals
MDT: Multidisciplinary team
PAG: Patient advocacy group
