# Supplementary material for: Optimising care and follow-up of adults with achondroplasia
Source: Orphanet J Rare Dis. 2022 Aug 20;17:318. doi: 10.1186/s13023-022-02479-3 (PMC9392284; doi:10.1186/s13023-022-02479-3)
Supplement: Supplementary file 1 — Additional file 1. Managing Achondroplasia into Adulthood – Healthcare Professionals Survey. [file 13023_2022_2479_MOESM1_ESM.pdf]

Managing Achondroplasia into Adulthood – HCP survey

Introduction

The European Achondroplasia Forum, a network of senior clinicians and orthopaedic surgeons from Europe and the Middle East representative of the achondroplasia clinical community, will be holding a workshop on 12th October to discuss the process of transition from paediatric to adult services and how achondroplasia is managed in adulthood. To establish current practices in Europe, and as a basis for discussion, we would welcome your feedback on how this is managed in your centre. The survey takes 12 minutes to complete.

A manuscript will be produced after the meeting, documenting the data from the survey, strategies, and recommendations from the meeting. If you would like to be acknowledged for your contribution, please include your name and centre below. This data will not be used for any other purposes. If you would like to attend the EAF meeting on the 12th October, please contact eaf@cesasmedical.com

1. Name (optional)

2. Centre (optional)

Objectives

- To establish current practice in your centre regarding the transition from paediatric to adult services and management of achondroplasia in adulthood
- To enable identification of barriers to effective care in adulthood across Europe and at a country/centre level
- To prompt discussion of best practice to enable recommendations to be made

Managing Achondroplasia into Adulthood – HCP survey

General questions

For the following questions, please answer questions based on practice in your centre. If you are aware of differences elsewhere in your country, please add a comment

* 3. Please select your speciality

- [ ] Paediatric endocrinologist
- [ ] Paediatrician
- [ ] Neuropaediatrician
- [ ] Neonatologist
- [ ] Obstetrician
- [ ] Orthopaedic surgeon
- [ ] Medical or clinical geneticist
- [ ] Genetic counsellor
- [ ] Rheumatologist
- [ ] GP
- [ ] Other (please specify)

Managing Achondroplasia into Adulthood – HCP survey

General questions

* 4. Does your centre treat:

- [ ] Paediatric patients?
- [ ] Adult patients?
- [ ] Both paediatric and adult patients?

EAF
European Achondroplasia Forum

Managing Achondroplasia into Adulthood – HCP survey

General questions

* 5. Which of the following best describes your centre?

- [ ] Academic institution
- [ ] General hospital
- [ ] Specialist achondroplasia centre
- [ ] Primary care
- [ ] University/teaching hospital
- [ ] Other (please specify)

6. Please add more details (optional)

Managing Achondroplasia into Adulthood – HCP survey

General questions

* 7. Approximately how many individuals with achondroplasia does your centre follow?

Paediatrics

Adults

Individuals moving from paediatric to adult services

8. Please add more details (optional)

Managing Achondroplasia into Adulthood – HCP survey

Transition from paediatric to adult services

* 9. At what age do patients transition from paediatric to adult services?

- <16 years
- 16–17 years
- 18 years
- 19 years+
- Other (please specify)

10. Please add more details (optional)

Managing Achondroplasia into Adulthood – HCP survey

Transition from paediatric to adult services

* 11. Is there a structured transition process from paediatric to adult services?

- [ ] Yes
- [ ] No
- [ ] I don't know

12. If yes, please describe the process

13. If yes, for how many patients is this process followed?

0% 100%

14. Please add more details (optional)

Managing Achondroplasia into Adulthood – HCP survey

Transition from paediatric to adult services

Approximately what proportion of patients are lost to follow-up at each of the following stages? (Please indicate as a percentage of total achondroplasia patients)

* 15. Prior to cessation of paediatric services

0% 50% 100%

* 16. During transition between paediatric and adult services

0% 50% 100%

* 17. At the point of cessation of paediatric services

0% 50% 100%

* 18. After access to adult services has commenced

0% 50% 100%

19. Please add more details (optional)

Managing achondroplasia in adulthood

If you do not manage adults, please answer to the best of your knowledge based on where your patients transition to after leaving your care

* 20. Which of the following best describes the centre where adults are treated?

- [ ] Academic institution
- [ ] Specialist achondroplasia centre
- [ ] University/teaching hospital
- [ ] Other (please specify)
- [ ] General hospital
- [ ] Primary care

21. Please give more details on the centre where adults are treated (optional)

Managing Achondroplasia into Adulthood – HCP survey

Managing achondroplasia in adulthood

* 22. Do patients leaving/attending your centre transition to an MDT for management of achondroplasia in adulthood?

- [ ] Yes
- [ ] No
- [ ] I don't know

Please add more details (optional)

EAF
European Achondroplasia Forum

Managing Achondroplasia into Adulthood – HCP survey

Managing achondroplasia in adulthood

* 23. Which specialties are part of an adult MDT managing achondroplasia?
Please select all that apply

- [ ] Endocrinologist
- [ ] Rheumatologist
- [ ] Clinical geneticist
- [ ] Orthopaedic surgeon
- [ ] Genetic counsellor
- [ ] Pulmonologist
- [ ] ENT specialist
- [ ] Obstetrician/gynaecologist
- [ ] Psychologist
- [ ] Rehabilitation and physical therapy specialist
- [ ] Pain consultant
- [ ] Nutritionist
- [ ] Dentist
- [ ] GP
- [ ] Physiotherapist
- [ ] Occupational therapist
- [ ] Social worker
- [ ] I don't know

Please add more details or additional specialties (optional)

EAF · European Achondroplasia Forum

Managing Achondroplasia into Adulthood – HCP survey

Managing achondroplasia in adulthood

* 24. Is there a lead clinician in the management of adults with achondroplasia?

- [ ] Yes
- [ ] No
- [ ] I don't know
- [ ] Please add more details (optional)

[ ]

25. If yes, who is the lead clinician?

- [ ] Endocrinologist
- [ ] Rheumatologist
- [ ] Clinical geneticist
- [ ] Orthopaedic surgeon
- [ ] Other (please specify)
- [ ] Genetic counsellor
- [ ] GP
- [ ] I don't know

[ ]

26. Please add more details (optional)

[ ]

Managing Achondroplasia into Adulthood – HCP survey

Managing achondroplasia in adulthood

* 27. Which specialities are most often required by adults with achondroplasia?

- [ ] Endocrinologist
- [ ] Rheumatologist
- [ ] Clinical geneticist
- [ ] Orthopaedic surgeon
- [ ] Genetic counsellor
- [ ] Pulmonologist
- [ ] ENT specialist
- [ ] Obstetrician/gynaecologist
- [ ] Psychologist
- [ ] Rehabilitation and physical therapy specialist
- [ ] Pain consultant
- [ ] Nutritionist
- [ ] Dentist
- [ ] GP
- [ ] Physiotherapist
- [ ] Occupational therapist
- [ ] Social worker
- [ ] Other (please specify)

Barriers to effective management in adulthood

* 28. In your experience, what are the top barriers to effective transition from paediatric to adult services and management in adulthood?
Please select up to five answers

- [ ] There is no MDT service available for adults
- [ ] Adult MDT services are not as experienced in achondroplasia management as the paediatric MDT
- [ ] The transition processes are unclear and challenging
- [ ] Lack of preparation for attending adult hospital without parents
- [ ] Individuals are lost to follow up in paediatric services
- [ ] Individuals are lost to follow up at the point of transition to adult services
- [ ] Lack of trust/relationship with new physician or team
- [ ] Other (please specify)
- [ ] Poor communication between the individual with achondroplasia (or family) and the MDT
- [ ] Poor communication between healthcare services
- [ ] Lack of interest/resistance from the individual with achondroplasia to access care
- [ ] Fewer needs for care
- [ ] Travel distance to the centre
- [ ] I don't know

29. Please add more details (optional)

Thank you!

Thank you for taking the time to complete the survey. Your responses are very important to us!
