# Supplementary material for: Optimising care and follow-up of adults with achondroplasia
Source: Orphanet J Rare Dis. 2022 Aug 20;17:318. doi: 10.1186/s13023-022-02479-3 (PMC9392284; doi:10.1186/s13023-022-02479-3)
Supplement: Supplementary file 2 — Additional file 2. Managing Achondroplasia into Adulthood – Patient Survey. [file 13023_2022_2479_MOESM2_ESM.pdf]

Managing Achondroplasia into Adulthood – Patient Survey

Introduction and objectives

The European Achondroplasia Forum, a network of senior clinicians and orthopaedic surgeons from Europe and the Middle East representative of the achondroplasia clinical community, will be holding a workshop on 12th October to discuss the process of transition of care from paediatric to adult services and how achondroplasia is managed in adulthood. To establish current practices in Europe and as a basis for discussion, we would welcome your feedback.

Please complete this survey from your perspective as a patient advocacy group representative. The survey takes no more than 10 minutes to complete.

Results of the survey will be presented during the EAF *Managing Achondroplasia into Adulthood* workshop on 12th October. If you would like to attend the workshop, please contact eaf@cesasmedical.com

Objectives

- To establish patient experience of the transition of care from paediatric to adult services and management of achondroplasia in adulthood
- To identify barriers to effective care into adulthood
- To establish what considerations are important to individuals with achondroplasia during the process of transition to adult services and in relation to management in adulthood

* 1. Please enter your country

Structure of achondroplasia care

Please always refer to your own country

* 2. To the best of your knowledge, how many centres of excellence/referral centres for skeletal dysplasia/rare bone conditions (including achondroplasia) are there in your country?

- [ ] 0
- [ ] 1
- [ ] 2
- [ ] 3
- [ ] ≥4
- [ ] I don't know

Please add more details (optional)

Structure of achondroplasia care

* 3. Are paediatric multidisciplinary teams for achondroplasia available in these centres of excellence/referral centres?

- [ ] Yes, in all
- [ ] Yes, in some
- [ ] No
- [ ] I don't know

Please add more details (optional)

Structure of achondroplasia care

* 4. Are adult multidisciplinary teams for achondroplasia available in these centres of excellence/referral centres?

- [ ] Yes, in all
- [ ] Yes, in some
- [ ] No
- [ ] I don't know

Please add more details (optional)

Transition of care from paediatric to adult services

* 5. Do healthcare teams or processes to facilitate the transition of children with achondroplasia from paediatric to adult services exist in your country?

- [ ] Yes, in all centres
- [ ] Yes, but only in centres of excellence/referral centres
- [ ] Not that I know of

Please add more details (optional)

Transition of care from paediatric to adult services

* 6. How well do you think the process of transition of care from paediatric to adult services is coordinated by healthcare providers in your country?

- [ ] Very well
- [ ] Well
- [ ] Satisfactorily
- [ ] Not satisfactorily
- [ ] Poorly
- [ ] I don't know

Please add more details (optional)

Transition of care from paediatric to adult services

* 7. How would you describe the overall patient experience of the transition of care from paediatric to adult services?

- [ ] Very good: well organised with excellent continuity of care
- [ ] Good: organised with good continuity of care
- [ ] Acceptable: somewhat organised with some aspects of continued care
- [ ] Poor: poorly organised with poor continuity of care
- [ ] Very poor: no organisation and no continuity of care
- [ ] I don't know

Please add more details (optional)

Managing achondroplasia in adulthood

* 8. To the best of your knowledge, which of the following best describes the centre where adults are treated?

Please select all that apply

- [ ] Academic institution
- [ ] Specialist achondroplasia centre
- [ ] University/teaching hospital
- [ ] General hospital
- [ ] Primary care
- [ ] Other, please specify below

Please add more details (optional)

Managing achondroplasia in adulthood

* 9. On average, how long does it take for an adult with achondroplasia to access an appointment with the multidisciplinary team?

- ☐ <1 month
- ☐ 1–4 months
- ☐ 5–10 months
- ☐ 11–12 months
- ☐ >12 months
- ☐ There is no multidisciplinary team in my country

Please add more details (optional)

Managing achondroplasia in adulthood

* 10. Which specialities are most often required by the adults with achondroplasia that your organisation supports?

Please select three answers

- [ ] Our organisation doesn't support adults
- [ ] Endocrinologist
- [ ] Rheumatologist
- [ ] Clinical geneticist
- [ ] Orthopaedic surgeon
- [ ] Genetic counsellor
- [ ] Pulmonologist
- [ ] Ear, nose and throat specialist
- [ ] Obstetrician/gynaecologist
- [ ] Other (please specify)
- [ ] Psychologist
- [ ] Rehabilitation and physical therapy specialist
- [ ] Pain consultant
- [ ] Nutritionist
- [ ] Dentist
- [ ] General practitioner/Family doctor
- [ ] Physiotherapist
- [ ] Occupational therapist
- [ ] Social worker

Barriers to effective management in adulthood

* 11. In your opinion, what are the top barriers to effective transition of care from paediatric to adult services and management in adulthood?

Please select up to five answers

- ☐ There is no multidisciplinary team (MDT) service available for adults
- ☐ Adult MDT services are not as experienced in achondroplasia management as the paediatric MDT
- ☐ The transition processes are unclear and challenging
- ☐ Lack of preparation for attending adult hospital without parents
- ☐ Individuals are lost to follow up in paediatric services
- ☐ Individuals are lost to follow up at the point of transition to adult services
- ☐ Lack of trust/relationship with new physician or team
- ☐ Poor communication between the individual with achondroplasia (or family) and the MDT
- ☐ Poor communication between healthcare services
- ☐ Lack of interest/resistance from the individual with achondroplasia to access care
- ☐ Fewer needs for care
- ☐ Excessive travel distance to the centre
- ☐ Other (please specify)

Considerations for the future

* 12. In your experience, what are the most important considerations for individuals with achondroplasia during the transition of care from paediatric to adult services?

There is no word limit

Considerations for the future

* 13. In your opinion, what could improve the transition process from paediatric to adult services?

There is no word limit

Considerations for the future

* 14. In your opinion, what are the most important considerations for how individuals with achondroplasia are managed as an adult?

There is no word limit

Considerations for the future

* 15. In your opinion, what could facilitate adults to continue with routine monitoring, to avoid urgent appointments or delays in care?

There is no word limit

Thank you

Thank you for taking the time to complete this survey – your input is important to us.
